# Monitoring plant responses in field-grown peanuts exposed to exogenously applied chitosan under full and limited irrigation levels

**DOI:** 10.1038/s41598-024-56573-6

**Published:** 2024-03-15

**Authors:** Kassem A. S. Mohammed, Hussein Mohamed Hussein, Ayman M. S. Elshamly

**Affiliations:** 1 Institute of African and Nile Basin Countries Research and Studies, Aswan University, Aswan, Egypt; 2 Water Studies and Research Complex. National Water Research Center, Cairo, Egypt

**Keywords:** Legumes, Mixed drought-alkali stresses, Abiotic stresses, Plant stress responses, Environmental impact, Fertilization

## Abstract

In recent decades, numerous studies have examined the effects of climate change on the responses of plants. These studies have primarily examined the effects of solitary stress on plants, neglecting the simultaneous effects of mixed stress, which are anticipated to transpire frequently as a result of the extreme climatic fluctuations. Therefore, this study investigated the impact of applied chitosan on boosting the resistance responses of peanuts to alkali and mixed drought-alkali stresses. Peanuts were grown in mid-alkaline soil and irrigated with full irrigation water requirements (100%IR), represented alkali condition (100% IR × alkali soil) and stress conditions (70% IR × alkali soil—represented mixed drought-alkali conditions). Additionally, the plants were either untreated or treated with foliar chitosan. The study evaluated various plant physio-chemical characteristics, including element contents (leaves and roots), seed yield, and irrigation water use efficiency (IWUE). Plants that experienced solitary alkali stress were found to be more vulnerable. However, chitosan applications were effective for reducing (soil pH and sodium absorption), alongside promoting examined physio-chemical measurements, yield traits, and IWUE. Importantly, when chitosan was applied under alkali conditions, the accumulations of (phosphorus, calcium, iron, manganese, zinc, and copper) in leaves and roots were maximized. Under mixed drought-alkali stresses, the results revealed a reduction in yield, reaching about 5.1 and 5.8% lower than under (100% IR × alkali), in the first and second seasons, respectively. Interestingly, treated plants under mixed drought-alkali stresses with chitosan recorded highest values of relative water content, proline, yield, IWUE, and nutrient uptake of (nitrogen, potassium, and magnesium) as well as the lowest sodium content in leaves and roots. Enhances the accumulation of (N, K, and Mg) instead of (phosphorus, calcium, iron, manganese, zinc, and copper) was the primary plant response to chitosan applications, which averted severe damage caused by mixed drought-alkali conditions, over time. These findings provide a framework of the nutrient homeostasis changes induced by chitosan under mixed stresses. Based on the findings, it is recommended under mixed drought-alkali conditions to treat plants with chitosan. This approach offers a promising perspective for achieving optimal yield with reduced water usage.

## Introduction

The southern region of Egypt is situated in a hyper-arid zone, which is distinguished by summertime temperatures that consistently surpass 40 °C^1^ and lacks any annual precipitation^2^. Moreover, the lands in this region are predominantly sandy and frequently exhibit various issues related to inadequate nutrient availability and water retention^3,4^. Furthermore, the induction of alkali stress via NaHCO_3_ and Na_2_CO_3_ results in additional stress^5^. Consequently, elevated temperatures, alkalinization, evaporation, and nutrient deficiency can be detrimental to plant development. The improvement of these alkali soils by the reclamation process can be achieved through various techniques. These techniques encompass the application of adequate quantities of high-quality water in well-drained conditions, the leaching process utilizing soluble calcium to substitute sodium^6,7^, and the development of salt-tolerant crop varieties^8^. However, the implementation of breeding as a remedy has thus far yielded only modest results in terms of creating tolerant crop varieties^9^. Likewise, in numerous regions across the globe, including Egypt, the acquisition of alternative reclamation techniques is hindered by the scarcity of freshwater resources. Certain farmers have been compelled to deviate from the optimal reclaim recommendations that ought to be attained by decreasing the quantity of water utilized for leaching. Hence, under these circumstances, salts accumulate in the rhizosphere as a result of inadequate water amounts and high evaporation rates^9^, which has numerous negative effects on plant growth due to salt and drought stresses^10^. Therefore, there is a need for more research to make land reclamation more sustainable in similar environments.

In this respect, the present study examines the viability of peanut plants as an additional reclaim method that can be maintained under drought-mild alkali stress conditions (with a salt content of no more than 3% and a pH range of 7.1–8.5)^5^. Given its relatively high drought tolerance, peanuts are capable of thriving in a variety of challenging environments^11^. Under drought or alkali environments, peanut has the potential to improve soil macronutrients. In this concern, Kebede^12^ demonstrated that as a legume species, peanut has distinctive effects on nitrogen assimilation via symbiotic relationships with microorganisms, including rhizobia, which fix atmospheric nitrogen and make it accessible to the roots for absorption. Moreover, peanuts have been found to enhance phosphorus and potassium availability under drought- alkali conditions^13^. However, Gentili et al.^14^ indicate that the availability of the majority of macronutrients is not the primary issue in alkaline soils, where it is increased. On the contrary, the availability of micronutrients, particularly iron (Fe), manganese (Mn), and zinc (Zn), exhibited a general decline. The reduced concentrations of these elements have the potential to detrimentally impact the growth of plants. Given the significant role that these nutrients play in alleviating the combined abiotic stressors^15,16^. Hence, in addition to the aforementioned advantages associated with planting peanuts in such conditions, prior research has demonstrated that peanut plants can improve the availability of micronutrients when irrigated^17,18^. Prior research by Duchene et al.^19^, Guinet et al.^20^ provided an explanation for this concern: peanut plants can increase nutrient absorption under normal irrigation conditions by increasing root exudates (ethylene release from their roots), which reduce soil acidity and improve the availability of insoluble soil nutrients^21^. However, the precise mechanism by which peanut plants improve their nutrient absorption in the presence of mixed drought and alkali stresses is still unknown. In typical growth conditions, numerous environmental stresses have a detrimental impact on the yield of several key crops^1,22^. Therefore, numerous plant species exhibit diverse molecular, physiological, and biochemical responses to distinct abiotic stresses. These include the synthesis of organic solutes, the closure of stomatal pores, the regulation of ion uptake by the roots, the accumulation of ions in particular plant organs (e.g., leaves), and the reduction of water potential in plant tissues^23–26^. Therefore, it is critical to acquire a thorough understanding of the monitoring of peanut responses in the presence of diverse stimuli. In a region where the achievement of that objective would safeguard more than 900 million hectares from drought-induced alkali damage^5^.

On the other hand, despite the drought tolerance of peanuts, the prolonged water stress that occurs during the flowering stage and pod filling continues to have detrimental effects on yield, as previously noted^27,28^. Therefore, the present study posits the hypothesis that when plants are under drought stress, they will exhibit a diminished capacity to absorb water and nutrients. Since the transportation of mineral nutrients from the soil to the organs of plants is intricately linked to water^29^. Additionally, when plants are under stress, they will employ specific mechanisms to tolerate such conditions^30,31^. In the interim, it is worth considering whether exogenous applications of chitosan would be more effective in improving water status within plant tissues. In this regard, chitosan (CH) has garnered significant attention as a viable strategy to mitigate the detrimental consequences of abiotic stress^32–34^. The utilization of antitranspirant materials, including CH, demonstrated significant potential in prolonging soil moisture retention, thereby delaying the advent of abiotic stress and ultimately improving IWUE^30,35,36^. A multitude of studies have identified a variety of advantages associated with the utilization of CH, such as improved plant transpiration, enhanced growth, chemical versatility, nutrient availability, and carboxylation efficiency in the presence of abiotic stressors^32,37,38^. Furthermore, the utilization of CH resulted in an increase in the content of photosynthetic elements, which in turn promoted stress tolerance^39^. Moreover, CH possesses a distinctive bioactive characteristic that can restrain serious injury to plants by stimulating their defense mechanisms^40^, when exposed to stressful conditions. Consequently, the implementation of CH may represent an optimal strategy for mitigating the adverse effects of drought-alkali combined stress^10,41^. However, the effects of CH applications on nutrient reorganization or enhancement, as well as on plant responses that could mitigate the detrimental effects of unfavorable conditions on peanut crops, remain poorly understood.

Drawing from the previously mentioned data, drought and alkali stress can occur concurrently in many regions of the globe, and they have severely impeded plant development in the agricultural sector worldwide. Nevertheless, the majority of research has primarily concentrated on the effects of individual types of stress, with relatively little emphasis placed on the consequences of drought-alkali mixed stress. Therefore, the current study aimed to A) Assess the effects of alkali and drought-alkali combined stress conditions on the growth, nutrient assimilation, carbohydrate content, chlorophyll, root-water content (RWC), proline, yield, and IWUE of peanuts. Also, this research was conducted to B) Examine the impact of CH application on nutrient homeostasis between the leaf and root of peanuts under conditions of mixed drought-alkali and alkali stress. And C) Assess the viability of improving the aforementioned parameters by implementing CH under these demanding conditions.

## Materials and methods

### The experimental site and design

The current experiment was conducted during the two summer seasons between May and September (2021/2022) at the experimental farm of Water Studies and Research Complex (WSRC), south of Egypt (22° 24' 11*''* N, 31°, 35' 43*''* E). The experimental area lies in arid climatic conditions. The average monthly data of maximum and minimum temperature, relative humidity, solar radiation, and wind speed were acquired from Toshka agrometeorological station during the consecutive cultivated seasons (2021/2022) presented in Table 1. The irrigation water came from a well was dug in the studied area with chemical characteristics are depicted in Table 2. Prior initiating the experiment, soil samples were taken from the topsoil layer (0–60 cm), right next to the plant (between 5 and 10 cm from the plant). The physical and chemical properties of the experimental soil were determined according to Estefan et al.^42^ and are presented in Table 3. To fulfill the purpose of the current study, the experiment layout was conducted in a split-plot arrangement on the basis of a randomized complete block design with four replications. The main plots were assigned to irrigation levels, while sub-plots were dedicated to the two foliar applications. The experiment consisted of 4 treatments and 16 experimental plots. The net space (10.0 m long × 4 m width) of each experimental unit. Two irrigation water levels, i.e., 100 and 70% of the peanut irrigation water requirement were evaluated in the current study, denoted IR100 and IR70.

**Table 1:** The average weather data from the experimental site throughout the period (May to September) during the 2021/2022 growing seasons.

	Temperature (C°)		Average Relative humidity (%)	Corrected E pan (mm day^−1^)	Wind speed (MS^−1^)	SR (watt m^−2^)
	Max	Min				
May	39.7 ± 0.20	22.2 ± 0.20	15.2 ± 0.21	9.6 ± 0.22	3.2 ± 0.22	293.7
June	41.7 ± 0.20	24.5 ± 0.21	16.6 ± 0.22	10.2 ± 0.24	3.6 ± 0.24	316.1
July	43.2 ± 0.19	24.8 ± 0.21	17.5 ± 0.20	10.3 ± 0.21	2.7 ± 0.25	306.7
August	42.4 ± 0.20	25.7 ± 0.20	19.2 ± 0.21	10.5 ± 0.22	2.9 ± 0.25	261.9
September	40.1 ± 0.21	23.7 ± 0.20	22.2 ± 0.24	9.9 ± 0.22	3.2 ± 0.21	239.6

*Max* maximum temperature, *Min* minimum temperature, *MS*^*−1*^ meter/second, *SR* solar radiation, and *mm* millimeter. The meteorological data were obtained from Toshka agrometeorological station, Egypt. Values are the mean of replicates ± standard errors.

**Table 2:** Water chemical properties at the experimental site, Egypt during the growing seasons 2021–2022.

Parameter	Unit	Value	Reference
pH		6.49 ± 0.72	Estefan et al.^42^
TDS	mg L^−1^	373.0 ± 0.71	
Calcium cations (Ca^2+^)	mg L^−1^	49.0 ± 0.73	
Magnesium cations (Mg^2+^)	mg L^−1^	10.0 ± 0.72	
Sodium cations (Na^+^)	mg L^−1^	88.0 ± 0.71	
Potassium cations (K^+^)	mg L^−1^	4.0 ± 0.72	
Chloride anions (Cl^−^)	mg L^−1^	109.0 ± 0.72	
Sulfate anions (SO_4_^2−^)	mg L^−1^	95.0 ± 0.71	

Each value represents the mean of replications ± standard errors.

*TDS* total dissolved solids.

**Table 3:** The physicochemical properties of the soil at the experimental site,

Parameter	Unit	Soil depth (cm)		Reference
		0–30	30–60	
Mechanical analysis				Estefan et al.^42^
Sand	% by weight	65.43 ± 0.71	67.2 ± 0.71	
Silt	% by weight	29.7 ± 0.71	30.26 ± 0.70	
Clay	% by weight	4.87 ± 0.70	2.54 ± 0.70	
Texture		Sandy Loam	Sandy Loam	
Chemical analysis				
pH		8.03 ± 0.70	8.13 ± 0.74	
ECe	ds m^−1^	4.10 ± 0.71	3.95 ± 0.70	
CaCO_3_	% by weight	11.7 ± 0.70	11.33 ± 0.73	
Available nitrogen	mg kg^−1^	28.1 ± 0.71	30.1 ± 0.70	
Available phosphorus	mg kg^−1^	7.5 ± 0.72	7.0 ± 0.74	
Available potassium	mg kg^−1^	92.5 ± 0.70	84.7 ± 0.71	
Available iron	mg kg^−1^	3.2 ± 0.71	3.8 ± 0.71	
Available manganese	mg kg^−1^	1.2 ± 0.70	1.7 ± 0.71	
Available zinc	mg kg^−1^	1.1 ± 0.72	1.7 ± 0.71	
Available copper	mg kg^−1^	0.1 ± 0.72	0.2 ± 0.71	
Available boron	mg kg^−1^	0.8 ± 0.72	0.9 ± 0.71	
CEC	mg 100 g^−1^	14.6 ± 0.72	14.82 ± 0.70	
Organic matter	% by weight	0.13 ± 2.0	0.15 ± 2.1	

Egypt in 2021–2022.

Each value represents the mean of replications ± standard errors.

Based on the chemical properties, the soil pH was alkaline (mean pH = 8.08). Therefore, the IR100 plot represented (alkali conditions), and the plants were irrigated by the full irrigation requirements. While, IR70 plot was represented (mixed drought-alkali stresses conditions), whereas the plants were grown in alkaline soil and irrigated by the limited irrigation requirements. The IR70 was chosen based on a previous study was tested various irrigation regimes on peanut crops in the same climatic conditions^17^.

The subplots were divided into two groups, namely control and CH. After six weeks of emergence, peanut plants were pretreated by foliar applications of CH (500 mg L^−1^), which was purchased from Alpha Chemika Co. The molecular weight of this product is (6 × 10^3^ kDa), deacetylation (82.4%), solubility (97% in 1% acetic acid), and viscosity (3275 cps). The CH solution was prepared by following a standardized method^43^ where: A solution of chitosan (5%) was prepared by adding high molecular weight of CH to 5% V/V glacial acetic acid solution on stirring at a pace of 500 rpm for 12 h on hot plate stirrer (ARE Heating Magnetic Stirrer). To stabilize the aqueous and to plays as a sticking agent in each spray, two drops of the tween 80 (polysorbate 80) were added, pH of CH solution was adjusted to 4.5 by adding 0.5 M NaOH and then filtered. Plants were sprayed with distilled water as a foliar application in the control treatment. The Foliar application of CH was applied four times (30, 45, 60, and 75 days), after the sowing date. All sprays for [CH—distilled water (control)] were performed by a hand pressure sprayer. Previous studies were used to determine the appropriate concentration range of CH (500 mg L^−1^)^44,45^.

### Planting operations

The peanut seeds (CV Giza 6) were obtained from the agricultural research center, Egypt. Before planting, peanut seeds were inoculated with the symbiotic N_2_—fixing specific rhizobium bacteria (*Rhizobium japonicum strains*) which was prepared by the Soil Microbiology Dept., Soil, Water and Environment Res. Inst., Agricultural Research Center (ARC), Egypt. The seeds were recommended as a high-yielding commercial cultivar. Moreover, these seeds and the implemented methods in the current study complied with international, national, and institutional guidelines and legislation. The coating seeds process was carried out using Arabic gum solution as a sticking agent^46,47^. As recommended by the Egyptian agriculture ministry, two seeds were sown in holes on May 5 and May 10 in the first and second growing seasons, respectively, at a rate of 120 kg seeds ha^−1^. Each sub-plot contained four planting rows that were 10 m in length with a spacing of 75 cm between the rows. A peanut plant density of 160,000 plants ha^−1^ was attained by sowing at a distance of 25 cm between plants on the rows. During the growing seasons, all agriculture practices were applied according to the recommendations set by the Ministry of Agriculture. Where nitrogen was applied as ammonium nitrate (33.5% N) at a level of 322 kg ha^−1^. This amount was divided into 6 equal doses, applied after 15 days of the sowing date; and repeated every 3–4 days with a rate of 60 kg ha^−1^ per portion till the flowering stage. Phosphorus in the form of superphosphate (15.5% P_2_O_5_) was added at a level of 715 kg ha^−1^, in one dose before planting. Potassium in the form of potassium sulfate (48% K_2_O) was added in two equal portions at a level of 180 kg ha^−1^ before and during the flowering growth stage. On the other hand, during the study period, no pesticides were applied.

### Water requirements assessment

#### Irrigation system

After full emergence and standardization of the numbers of peanut plants per plot at (960 plants plot^−1^), peanut plants were irrigated following the two irrigation water levels. A set sprinkler irrigation system was used to irrigate peanut plants. Each irrigation plot was equipped with a manometer valve to control the operating pressure at 2.5 bar. Additionally, the plots were equipped with a flow emitter to control the mounts of the targeted irrigation water level. Moreover, to avoid interactions between plots there was a distance of 9 m buffer zones.

#### *Crop evapotranspiration (*ETc*)*

The soil moisture parameters were measured before the experiment was initiated. After that, the reductions in the soil moisture were recorded, till it reaches to 45% of the available water, which a previous study indicated it was the critical limit on yield^48^. Accordingly, based on this knowledge the irrigation event was set every 2 days. The amounts of water requirement were calculated by the CROPWAT, version 8.0^49^ (a computer program for irrigation planning and management) combined with a climatic database those obtained from Toshka agrometeorological station. Taking into consideration that the reference evapotranspiration of peanuts (ETo) was determined based on Fao Penman–Monteith, following the recommendations of Allen et al.^50^. ETc for peanuts was determined according to Waller & Yitayew^51^ as follows:

$${\text{ETc }} = \, \left( {{\text{ETo }} \times {\text{ Kc}}} \right),$$

where ETc = crop evapotranspiration (mm day^−1^), ETo = reference evapotranspiration (mm day^−1^), Kc = crop coefficient, dimensionless, (was equaled 0.60, 0.76, 0.88, 0.78, and 0.63 for Kc _initial_, Kc _development_, Kc _mid_, Kc _late_, and Kc _harvest_ according to Zayton et al.^52^.

Each plot received amounts of irrigation water, calculated according to Elshamly^53^ as follows:

$$\mathrm{IR }=\frac{[\left({\text{Etc}}\times {\text{Dd}}\right)+{\text{Lf}}]}{{\text{Es}}}$$

where: IR = calculated irrigation water requirements (m^3^ ha^−1^), Etc = crop evapotranspiration, Lf = leaching water requirement 10%, Dd = time intervals, Es = irrigation system efficiency, 0.78.

The amount of IR70 treatment was proportionally obtained from the IR100. The peanut plants were irrigated with limited irrigation level only in the development and flowering growth stages. Accordingly, the average total seasonal amounts of IR were 8522 and 7075 m^3^ ha^−1^ for the IR100 and IR70, respectively.

## Chemical and physiological measurements

### After 65 days of sowing date

#### Relative water content (RWC)

##### Nature and number of the samples

In order to measure the RWC of peanut, ten plants in each plot were taken. Then one fully expanded leaf was taken from each plant resulting in 10 leaves per plot, and repeated four times.

##### The measurement

The leave samples were detached and immediately weighed to determine fresh weight (FW). After re-hydration for 24 h at 25 °C in darkness chamber, turgid weight (TW) was determined. Then, after oven-drying in 75 °C for 72 h dry weight (DW) was recorded. RWC ratio calculated based on Ref.^26^ as follows:

$${\text{RWC}}=\frac{{\text{FW}}-{\text{DW}}}{{\text{TW}}-{\text{DW}}}\times 100$$

#### Determination of total chlorophyll content, total carbohydrates, and proline

##### Nature and number of the samples

From ten different plants, located in the middle of the plot, the upper three leaves of the main stem of each peanut were collected, resulting in 30 leaves per plot, and repeated four times. After removing the leaves from the selected plant, the total chlorophyll content, total carbohydrates and proline contents were measured as follows:

##### The measurement of total chlorophyll content

In the first, 0.2 g of peanut leaf samples were dried and powdered. Then the samples were added to ethanol (80%), homogenized, and stirred for 3 h. The solvent was evaporated, after that the residues was mixed with distilled water and is ready for the following analysis^54^. Then, the total chlorophyll determination as per El-Saadony et al.^55^. To measure the total chlorophyll, the extractor was centrifuged at 10,000 × g for 5 min followed by the removal of debris. The absorbance of the supernatant was measured by using a spectrophotometer at 645 and 663 nm for chlorophyll (a and b) respectively. The average total chlorophyll expressed as (μg g^−1^) was obtained as follows:

$$\mathrm{Total \,chlorophyll }=\mathrm{chlorophyll \,a }+\mathrm{chlorophyll \,b}$$

##### The measurement of total carbohydrates content

Total carbohydrates expressed as (%), were determined by standard calibration curve of glucose method as described by El-Katony et al.^56^. Where, 0.5 g of the peanut leaf samples were air-dried and ground into a powder and then the aliquots were mixed with anthrone reagent (8.6 mM anthrone in 80% v/v H_2_SO_4_). Then on a water bath at 80 °C for 10 min, the mixture was boiled, then cooled for 30 min on the ice. Finally, by using a standard calibration curve of glucose, the absorbance was measured at 623nm.

##### The measurement of proline content

To measure proline in extracts, 0.5 g of fresh leaves sample were grounded in a mortar using liquid nitrogen and pestle with 10 ml of sulfosalicylic acid and filtered. Then, the rest procedures were done by following standardized method as described by Sahin^57^. Where the extract was homogenized in a water bath at 99 °C, then the solution was stored in the refrigerator until the extraction process. Then the supernatant was centrifuged at 12,000 × g for 10 min. 2 ml of supernatant was taken and mixed with both (2 ml of glacial acetic acid and 3 ml of 2.5% ninhydrin reagent). After that the reaction mixture was incubated in boiling water for 60 min. Then it was extracted by adding 4 ml of toluene. The proline concentration in the absorbance was determined using a spectrophotometer at 520 nm, and the following formula:

$${\text{Proline}}=\frac{\mathrm{Toluene }\left({\text{ml}}\right)\times \mathrm{ proline }({\text{mg}}/{\text{ml}})}{115.13\mathrm{ ml}/{\text{mol}}}\times \frac{\mathrm{ samples }({\text{g}})}{5}$$

### At the harvest

#### Measuring the morphological characteristics

The plots were harvested entirely by hand. First, ten plants per plot were randomly selected from the middle two rows at harvesting date (as a subsample). After removing the pods from the selected plant, the shoot fresh weight of selected plants was measured using a scale, then the results were recorded. At the same time the root length was recorded and the pods from the selected ten plants were air-dried. For the determination of seed index (10 seeds weight), the seeds were separated from the pods, then 10 seeds samples were taken and recorded as the mean of samples taken at random from the ten harvested plants of each treatment. For the calculation of pods weights, in each plot (random ten samples with 1 m^2^) was harvested manually, air-dried, and the weight of the pods was determined on a scale when the moisture content was (8% approximately). Then the results were expressed as pod yield per m^2^, which was then converted to kg per hectare.

#### Measuring nutrient content in the shoot and roots

The dried peanut samples of ten different peanut plants (leaves and roots) were collected, weighed and ground into a fine powder. The nitrogen (N) content was measured by weighing 0.5 g of the fine powder and transfer quantitatively into a 100 ml digestion tube, then N content was measured by the Kjeldahl method according to Estefan et al.^42^. Phosphorus (P) contents were measured using the colorimetry analysis, where in a volumetric flask with (sulfuric acid, salicylic acid, and hydrogen peroxide), the plant sample (leaves and roots) was digested. After providing the extract, it was measured by using a spectrophotometer at 470 nm wavelength^42^. The potassium (K) and sodium (Na) contents of the dry digestion extracts for leaves and roots were analyzed using a flame -photometer as described by Shekari et al.^58^. Where in a furnace (550 °C) and dissolving in 0.5-M HCl the dry digestion extracts were prepared, then filtered and diluted to a certain volume. On the other hand, by digesting 100 mg of the fine powder in a mixture of concentrated nitric acid and perchloric acid (3:1; v/v) at 175 °C. Magnesium (Mg) and calcium (Ca) contents were measured using an atomic absorption spectrophotometer, as outlined by Estefan et al.^42^. While, the minerals contents (iron (Fe), zinc (Zn), manganese (Mn), and copper (Cu) were estimated according to Lukic´ et al.^59^. Where the plant organs (leaves–roots) were sieved and became ash at 550 °C and then dissolved in 2-M HCl and filtered through a filter paper and diluted to a certain volume. After that, a digestion was used, and by using the atomic absorption spectrophotometer, (Fe, Zn, Mn, and Cu) were measured.

#### Measurements of soil pH

In each subplot, six soil cores of 10 cm diameter from the middle two rows, were sampled down to 0.40 cm, then mixed thoroughly in the field to form one composite sample per subplot. The soil samples (2 mm diameter) were used to determine soil pH by potentiometric method (digital ionalyzer/501, Orion research multifunctional pH meter), and the soil: water ratio was 2.5:1.

#### Irrigation water use efficiency (IWUE) calculation

The IWUE was calculated as described by^60^ as follows: For the IWUE determination in both growing seasons, crop yield ratio (Y) to total irrigation water requirements in the field, was calculated and recorded under each treatment.

### The statistical analysis

Statistical differences among irrigation levels and CH application in each growing season were analyzed and estimated using ANOVA tests. By CoStat (version 6.303) software program^61^, the differences between the mean values of irrigation levels, foliar chitosan application, and their interaction in each growing season were performed using the Tukey HSD post hoc tests at (p < 0.05), wherever found significant were also calculated and presented. To determine the variation in the measured variables obtained from different treatments, the descriptive statistics, such as the mean, standard deviation, and standard error, were calculated according to Casella et al.^62^. Different lowercase letters above error bars indicate statistically significant differences (p < 0.05).

## Results

### The solitary and interaction effects of CH and IR under alkali & mixed drought-alkali conditions on

#### RWC

**Figure 1:**
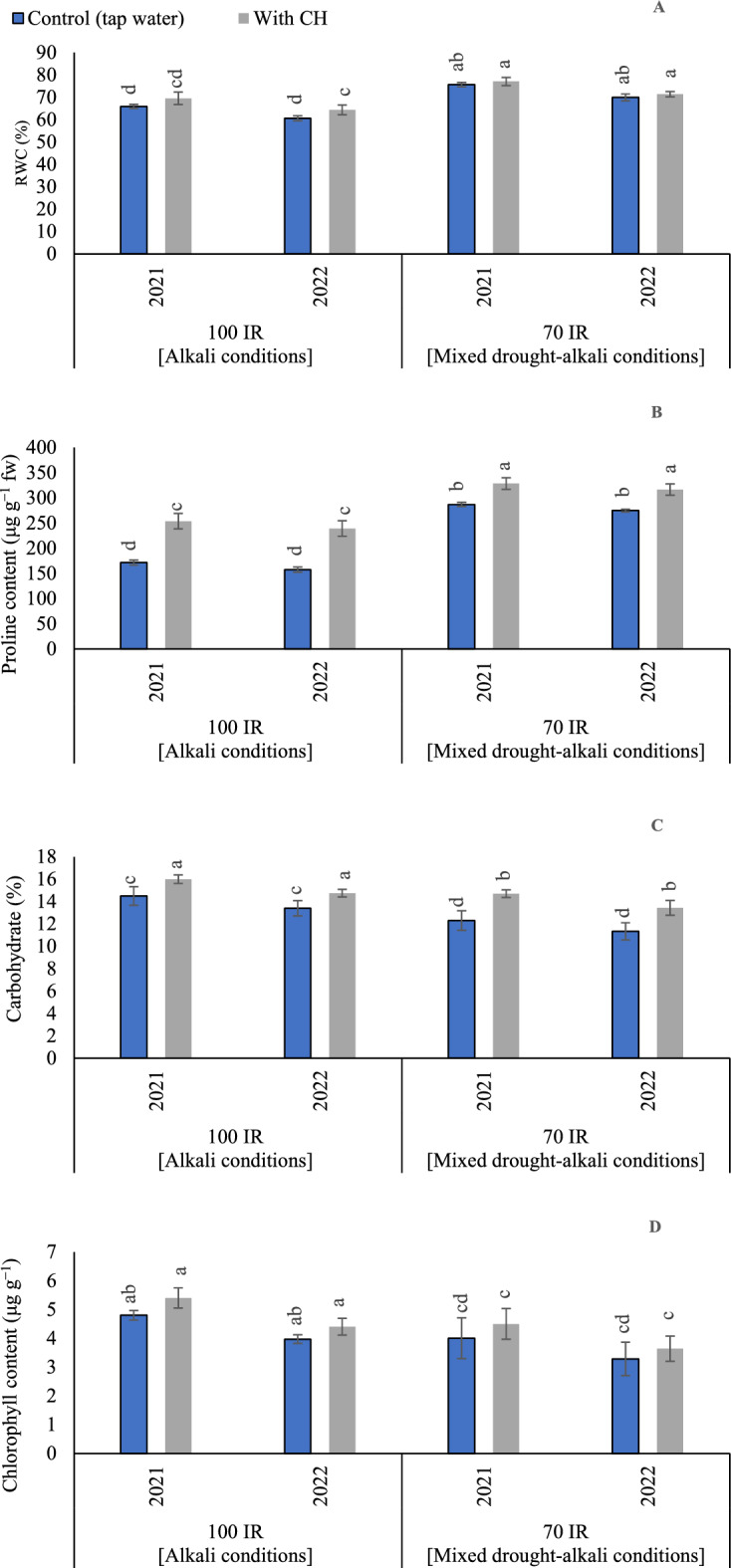
The solitary and interaction effects of CH and IR on (**A**) relative water content (RWC), (**B**) proline, (**C)** carbohydrate, and (**D**) total chlorophyll at two seasons of 2021/2022. Vertical bars represent ± standard error (SE) of the means. Different letters in the bars indicate significant differences between treatments at P ≤ 0.05 level by the Tukey’s test. Abbreviations: Control (tap water applications); CH (foliar chitosan applications at a rate of 500 mg L^−1^); IR100% (100% of the irrigation water requirements- represent alkali condition); IR 70% (70% of the irrigation water requirements- represent mixed drought-alkali conditions).

Based on the results of variance analysis (Tables 4 and 5), the sole effect of examined CH and IR was obviously affected RWC in the two growing seasons. The *p* values for CH were recorded (0.002) in the first and second seasons. On the side, *p* values for IR were recorded (0.029 and 0.001) in the first and second seasons, respectively. While the interaction between CH and IR had non-significant influence (*p* = 0.078) in the first season and significant influence (*p* = 0.033) in the second season. RWC was increased under 70% IR (mixed drought-alkali conditions) than 100% IR (alkali condition) in the first and second seasons, as shown in (Fig. 1A). By applying CH under 70% IR (mixed drought-alkali condition), the RWC ratio was enhanced by 16.8 and 17.7% compared to control (70%) IR in the first and second seasons, respectively. Likewise, by applying CH under 100% IR (mixed drought-alkali conditions), RWC ratio was enhanced by 5.6 and 6.1% compared to control (100% IR) in the first and second seasons, respectively. The highest RWC (77 and 71.3%) was recorded by applying foliar applications of CH under 70% IR (mixed drought-alkali conditions) in the first and second seasons, respectively. While, the lowest RWC (65.9 and 60.6%) was recorded under control 70% IR (alkali conditions) in the first and second seasons, respectively.

**Table 4:** Variance analysis of the investigated parameters in the first growing season (2021).

Source of variation	df	CHL	RWC	PROL	CAR	Y	Cu L	Cu R	Ca L
Irrigation levels (IR)	1	*	*	*	*	NS	*	*	*
Chitosan (CH)	1	*	*	*	*	*	*	*	*
IR × CH	1	NS	NS	*	*	*	*	NS	*
*p*-value									
		0.001	0.002	< 0.001	< 0.001	0.946	0.001	< 0.001	0.009
		0.026	0.029	< 0.001	< 0.001	< 0.001	0.003	< 0.001	< 0.001
		0.829	0.078	< 0.001	0.049	0.005	0.045	0.380	< 0.001

	Df	IWUE	NL	NR	PL	PR	KL	KR	Ca R
Irrigation levels (IR)	1	*	*	*	*	*	*	*	*
Chitosan (CH)	1	*	*	*	*	*	*	*	*
IR × CH	1	*	NS	*	*	*	*	*	*
*p*-value									
		< 0.001	0.004	0.002	< 0.001	0.004	0.005	0.008	0.049
		< 0.001	< 0.001	< 0.001	< 0.001	0.002	< 0.001	< 0.001	< 0.001
		0.002	0.108	0.026	0.006	0.033	0.003	0.006	< 0.001

	df	Na L	Na R	Mg L	Mg R	Fe L	Fe R	Mn L	Mn R
Irrigation levels (IR)	1	*	*	*	*	*	*	*	*
Chitosan (CH)	1	*	*	*	*	*	*	*	*
IR × CH	1	*	NS	*	*	NS	*	*	NS
*p*-value									
		< 0.001	0.001	< 0.001	< 0.001	0.001	< 0.001	0.008	0.003
		< 0.001	< 0.001	< 0.001	< 0.001	0.003	< 0.001	0.001	< 0.001
		< 0.001	0.097	< 0.001	< 0.001	0.074	0.002	0.024	0.051

	df	Zn L	Zn R						
Irrigation levels (IR)	1	*	*						
Chitosan (CH)	1	*	*						
IR × CH	1	*	*						
*p*-value									
		0.004	0.019						
		< 0.001	< 0.001						
		0.004	0.001						

*CHL* total chlorophyll, *RWC* relative water content, *PROL* proline, *CAR* carbohydrates, *NL* nitrogen in leaves, *NR* nitrogen in roots, *PL* phosphorus in leaves, *PR* phosphorus in roots, *Ca L* calcium in leaves, *KL* potassium contents in leaves, *KR* potassium contents in roots, *Na L* sodium in leaves, *Na R* sodium in roots, *Ca L* calcium in leaves, *Ca R* calcium in roots, *Y* peanuts yield, *IWUE* irrigation water use efficiency, *Fe R* iron in roots, *Fe L* iron in leaves, *Mn L* manganese in leaves, *Mn R* manganese in roots, *Zn L* zinc in leaves, *Zn R* zinc in roots, *Cu L* copper contents in leaves, *Cu R* copper contents in roots, *NS* non-significance.

*Significance at P ≤ 0.05.

**Table 5:** Variance analysis of the investigated parameters in the second growing season (2022).

Source of variation	df	CHL	RWC	PROL	CAR	Y	Cu L	Cu R	Mn R
Irrigation levels (IR)	1	*	*	*	*	NS	*	*	*
Chitosan (CH)	1	*	*	*	*	*	*	*	*
IR × CH	1	NS	*	*	*	*	*	NS	NS
*p*-value									
		0.006	0.002	< 0.001	0.004	0.114	0.044	0.013	0.003
		0.026	0.001	< 0.001	0.001	< 0.001	< 0.001	< 0.001	< 0.001
		0.820	0.033	< 0.001	0.046	0.017	< 0.001	0.109	0.056

	df	IWUE	NL	NR	PL	PR	KL	KR	Ca L
Irrigation levels (IR)	1	*	*	*	*	*	*	*	*
Chitosan (CH)	1	*	*	*	*	*	*	*	*
IR × CH	1	*	NS	NS	*	*	*	*	*
*p*-value									
		< 0.001	0.002	0.008	< 0.001	0.001	0.002	0.008	0.030
		< 0.001	< 0.001	< 0.001	< 0.001	< 0.001	< 0.001	< 0.001	< 0.001
		0.005	0.101	0.309	0.001	0.008	0.004	0.005	< 0.001

	df	Ca R	Na L	Na R	Mg L	Mg R	Fe L	Fe R	Mn L
Irrigation levels (IR)	1	*	*	*	*	*	*	*	*
Chitosan (CH)	1	*	*	*	*	*	*	*	*
IR × CH	1	*	*	*	*	*	*	*	*
*p*-value									
		0.046	0.001	0.020	0.001	< 0.001	0.001	< 0.001	< 0.001
		< 0.001	< 0.001	< 0.001	0.001	< 0.001	< 0.001	< 0.001	< 0.001
		< 0.001	0.001	< 0.001	0.001	< 0.001	0.001	0.041	0.050

	df	Zn L	Zn R						
Irrigation levels (IR)	1	*	*						
Chitosan (CH)	1	*	*						
IR × CH	1	*	*						
*p*-value									
		0.002	0.003						
		< 0.001	< 0.001						
		< 0.001	0.0002						

*CHL* total chlorophyll, *RWC* relative water content, *PROL* proline, *CAR* carbohydrates, *NL* nitrogen in leaves, *NR* nitrogen in roots, *PL* phosphorus in leaves, *PR* phosphorus in roots, *Ca L* calcium in leaves, *KL* potassium contents in leaves, *KR* potassium contents in roots, *Na L* sodium in leaves, *Na R* sodium in roots, *Ca L* calcium in leaves, *Ca R* calcium in roots, *Y* peanuts yield, *IWUE* irrigation water use efficiency, *Fe R* iron in roots, *Fe L* iron in leaves, *Mn L* manganese in leaves, *Mn R* manganese in roots, *Zn L* zinc in leaves, *Zn R* zinc in roots, *Cu L* copper contents in leaves, *Cu R* copper contents in roots, *NS* non-significance.

*Significance at P ≤ 0.05.

#### Proline content

Based on the results of variance analysis, the sole and interaction effect of CH and IR on proline content were significant (p < 0.05) in the two growing seasons. The *p* values for CH were recorded (< 0.001) in the first and second seasons. Also, *p* values for IR were recorded (< 0.001) in the first and second seasons. Moreover, the interaction between CH and IR recorded (*p* =  < 0.001) in the first season and second seasons. By comparing the treatments under alkali and mixed drought-alkali, the proline concentrations increased under the mixed drought-alkali conditions in the two growing seasons, as can be seen in (Fig. 1B). The obtained results showed that the highest proline concentrations were obtained by CH under mixed drought-alkali conditions. Where it relative to (CH × 100% IR) increased by (29.5 and 32.3%) under (CH × 70% IR) in the first and second seasons, respectively. While the lowest proline concentrations were recorded by applying tap water applications under 100% IR (alkali condition) in the two growing seasons.

#### Carbohydrate

Based on the results of variance analysis, the sole and interaction effect of CH and IR on carbohydrate content were significant (p < 0.05) in the two growing seasons. The *p* values for CH were recorded (< 0.001 and 0.004) in the first and second seasons, respectively. On the side, *p* values for IR were recorded (< 0.001 and 0.001) in the first and second seasons, respectively. While the interaction between CH and IR was recorded (*p* = 0.049) in the first season and (*p* = 0.046) in the second season. The effects of the CH applications and IR (100 and 70%), on the carbohydrate contents, are presented in (Fig. 1C). By comparing the treatments under alkali and mixed drought-alkali, the carbohydrate contents were significantly increased under the alkali conditions in the two growing seasons than the mixed drought-alkali conditions. Additionally, carbohydrate contents were significantly enhanced by applying CH applications. The obtained results showed that by applying CH applications under 100% IR (alkali condition), carbohydrate contents increased by (8.8 and 9.7%) compared to 70% IR (mixed drought-alkali conditions) in the first and second seasons, respectively. While, by adopting 100% IR without applying CH applications under 100% IR, carbohydrate contents increased by (17.9 and 18.3%) compared to control mixed drought-alkali treatment.

#### Total chlorophyll

Based on the results of variance analysis (Tables 4 and 5), the sole effect of examined CH and IR was affected the chlorophyll concentration significantly in the two growing seasons. The *p* values for CH were recorded (0.001 and 0.006) in the first and second seasons, respectively. On the side, *p* values for IR were recorded (0.026) in the first and second seasons. While the interaction between CH and IR had non-significant influence (*p* = 0.829) in the first season and (*p* = 0.820) in the second season. By comparing the treatments under alkali and mixed drought-alkali, total chlorophyll was significantly increased and attained the highest values under the alkali conditions in the two growing seasons compared to the mixed drought-alkali conditions (Fig. 1D). The results indicated that by applying CH applications under 100% IR (alkali condition), total chlorophyll contents enhanced by (20.0 and 20.9%) compared to 70% IR (mixed drought-alkali conditions) in the first and second seasons, respectively. While, by adopting 100% IR without applying CH applications under 100% IR, carbohydrate contents increased by (20.0 and 21.0%) compared to control mixed drought-alkali treatment.

#### N concentrations in peanut leaves

**Figure 2:**
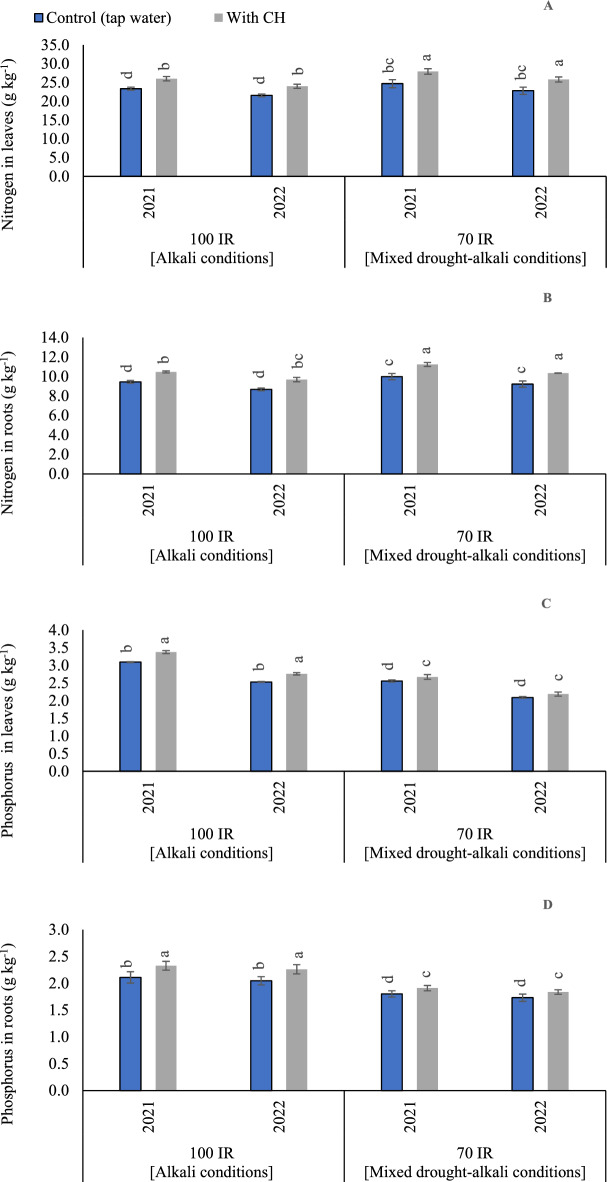
The solitary and interaction effects of CH and IR on (**A**) Nitrogen content in leaves, (**B**) Nitrogen content in roots, (**C**) and Phosphorus content in leaves, and (**D**) Phosphorus content in roots at two seasons of 2021/2022. Vertical bars represent ± standard error (SE) of the means. Different letters in the bars indicate significant differences between treatments at P ≤ 0.05 level by the Tukey’s test. Abbreviations: Control (tap water applications); CH (foliar chitosan applications at a rate of 500 mg L^−1^); IR100% (100% of the irrigation water requirements- represent alkali condition); IR 70% (70% of the irrigation water requirements- represent mixed drought-alkali conditions).

According to the ANOVA results, the sole influence of examined CH and IR on N content was significant (*p* < 0.05) in the two growing seasons. The *p* values for CH were recorded (0.004 and 0.002) in the first and second seasons, respectively. On the side, *p* values for IR were equaled and recorded (< 0.001) in the first and second seasons. While the interaction between CH and IR was recorded (*p* = 0.108 and 101) in the first season and second seasons, respectively. Relative to control full irrigation treatment (alkali condition), N concentrations increased by 5.6 and 5.5% for control limited irrigation treatment (mixed drought-alkali conditions), in the first and second seasons, respectively (Fig. 2A). Likewise, relative to control CH under full irrigation treatment (alkali condition), N concentrations increased by 7.7 and 7.5% for control CH under limited irrigation treatment (mixed drought-alkali conditions), in the first and second seasons, respectively. On the other side, the gained results showed that the maximum value of N contents was observed by applying foliar applications of CH under mixed drought-alkali conditions. While the lowest N concentrations were recorded by applying full irrigation requirements under alkali condition without CH applications.

#### N concentrations in peanut roots

Based on the results of variance analysis, the sole effect of examined CH and IR was significant in the two growing seasons. The *p* values for CH were recorded (0.002 and 0.008) in the first and second seasons, respectively. On the side, *p* values for IR were equaled and recorded (< 0.001) in the first and second seasons. While the interaction between CH and IR had a significant influence in the first season and had a non-significant influence in the second season. The *p* values for the interaction were (*p* = 0.108 and 0.026) in the first season and second seasons, respectively. The obtained results in (Fig. 2B) showed that the maximum N contents in the root were observed by applying foliar applications of CH under mixed drought-alkali conditions in the two growing seasons. While the lowest N concentrations in root were recorded by applying full irrigation requirements under alkali condition without CH applications.

#### P concentrations in peanut leaves

Based on the results of variance analysis, the sole and interaction effect of CH and IR on P contents in leaves were significant (p < 0.05) in the two growing seasons. The *p* values for CH were recorded (< 0.001) in the first and second seasons. Also, *p* values for IR were recorded (< 0.001) in the first and second seasons. While the interaction between CH and IR was recorded (*p* = 0.006) in the first season and (*p* = 0.001) in the second season. The obtained results in (Fig. 2C) showed that adopting stressful irrigation level (mixed drought-alkali conditions) without applying CH, led to significant decrease in P concentrations. Interestingly, treatment peanut plants with foliar applications of CH improved the P contents under alkali and mixed drought-alkali conditions. However, applying foliar application of CH under alkali conditions (70% IR), improved P concentrations better than mixed drought-alkali conditions. Therefore, treating peanut plants with CH applications and applying the full irrigation requirements under alkali conditions, could help the plants to pass the adverse impacts of alkali stress and attain higher P concentrations.

#### P concentrations in peanut roots

Based on the results of variance analysis, the sole and interaction effect of examined CH and IR on P contents in root were significant in the two growing seasons. The *p* values for CH were recorded (0.004 and 0.001) in the first and second seasons, respectively. On the side, *p* values for IR were recorded (0.002 and < 0.001) in the first and second seasons, respectively. While the interaction between CH and IR was recorded (*p* = 0.033) in the first season and (*p* = 0.008) in the second season. By comparing the treatments under alkali and mixed drought-alkali, the P contents in root were increased under alkali conditions in the two growing seasons. Relative to control limited irrigation treatment (mixed drought-alkali conditions), P contents increased by 16.7 and 17.7% for control full irrigation treatment (alkali conditions), in the first and second seasons, respectively (Fig. 2D). Likewise, relative to control CH under limited irrigation treatment (mixed drought-alkali conditions), P contents increased by 21.1 and 27.8% for control CH under full irrigation treatment (alkali conditions), in the first and second seasons, respectively.

#### K concentrations in peanut leaves

**Figure 3:**
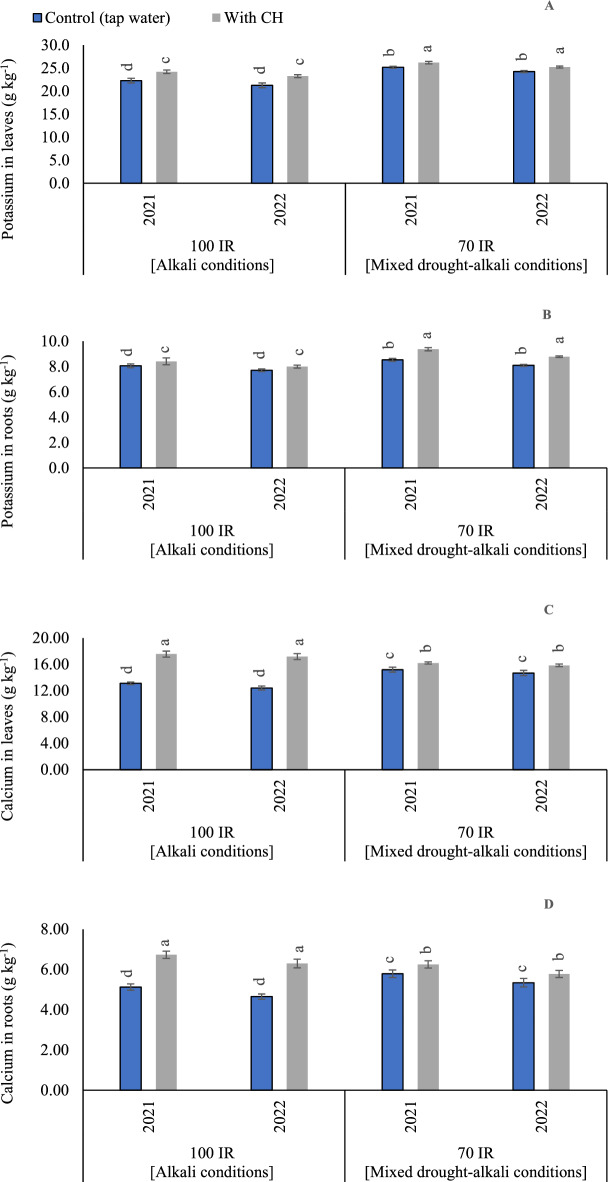
The solitary and interaction effects of CH and IR on (**A**) Potassium content in leaves, (**B**) Potassium content in roots, (**C**) and Calcium content in leaves, and (**D**) Calcium content in roots at two seasons of 2021/2022. Vertical bars represent ± standard error (SE) of the means. Different letters in the bars indicate significant differences between treatments at P ≤ 0.05 level by the Tukey’s test. Abbreviations: Control (tap water applications); CH (foliar chitosan applications at a rate of 500 mg L^-1^); IR100% (100% of the irrigation water requirements- represent alkali condition); IR 70% (70% of the irrigation water requirements- represent mixed drought-alkali conditions).

Based on the results of variance analysis, the sole and interaction effect of examined CH and IR on K contents in leaves were significant (p < 0.05) in the two growing seasons. The *p* values for CH were recorded (0.005 and 0.002) in the first and second seasons, respectively. On the side, *p* values for IR were recorded (< 0.001) in the first and second seasons. While the interaction between CH and IR was recorded (*p* = 0.003) in the first season and (*p* = 0.004) in the second season. As can be seen in (Fig. 3A), by comparing alkali and mixed drought-alkali conditions in the control treatments without applying CH applications, choosing limited-watered conditions (mixed drought-alkali conditions), results in significant increases for K concentrations in the peanut leaves in the two growing seasons. In this sense, when adopting control full irrigation treatment (alkali conditions), K content was decreased by 13.0 and 13.6% compared to control limited-watered conditions, in the first and second seasons, respectively. Likewise, relative to control CH under full irrigation treatment (alkali conditions), K contents decreased by 8.3 and 8.2% for control CH under limited irrigation treatment (mixed drought-alkali conditions), in the first and second seasons, respectively.

#### K concentrations in peanut roots

Based on the results of variance analysis, the sole and interaction impacts of examined CH and IR on K contents in root were significant in the two growing seasons. The *p* values for CH were recorded (0.008) in the first and second seasons. Also, *p* values for IR were recorded (< 0.001) in the first and second seasons. While the interaction between CH and IR was recorded (*p* = 0.006) in the first season and (*p* = 0.005) in the second season. Relative to control alkali conditions, K contents increased by 5.0 and 5.2% for control mixed drought-alkali conditions, in the first and second seasons, respectively (Fig. 3B). Likewise, relative to control CH under mixed drought-alkali conditions, K contents increased by 11.9 and 10.0% for control CH under alkali conditions, in the first and second seasons, respectively.

#### Ca concentrations in peanut leaves

According to the ANOVA results, the sole and interaction influence of CH and IR on Ca contents in roots were significant in the first and second seasons. The *p* values for CH were recorded (0.009 and 0.030) in the first and second seasons, respectively. On the side, *p* values for IR were recorded (< 0.001) in the first and second seasons. While the interaction between CH and IR was recorded (*p* < 0.001) in the first and second season. Relative to control CH under limited irrigation treatment (mixed drought-alkali conditions), Ca contents increased by 8.3 and 8.5% for control CH under full irrigation treatment (alkali conditions), in the first and second seasons, respectively (Fig. 3C). The highest Ca contents were significantly recorded by applying the foliar applications of CH under full irrigation treatment (alkali conditions), in the first and second seasons. On the other side, the lowest Ca contents were obtained by adopting full irrigation requirements under alkali or mixed drought-alkali conditions condition and applying tap water applications.

#### Ca concentrations in peanut roots

Based on the results of variance analysis, the sole and interaction influence of CH and IR on Ca contents in roots were significant in the first and second seasons. The *p* values for CH were recorded (0.049 and 0.046) in the first and second seasons, respectively. On the side, *p* values for IR were recorded (< 0.001) in the first and second seasons. While the interaction between CH and IR was recorded (< 0.001) in the first and second season. The obtained results in (Fig. 3D) showed that the maximum Ca contents in roots were observed by applying foliar applications of CH under alkali conditions in the two growing seasons. On the contrary, negative significant influence in the Ca contents were seen when the tap water was applied under the same growth conditions (alkali conditions).

#### Mg concentrations in peanut leaves

**Figure 4:**
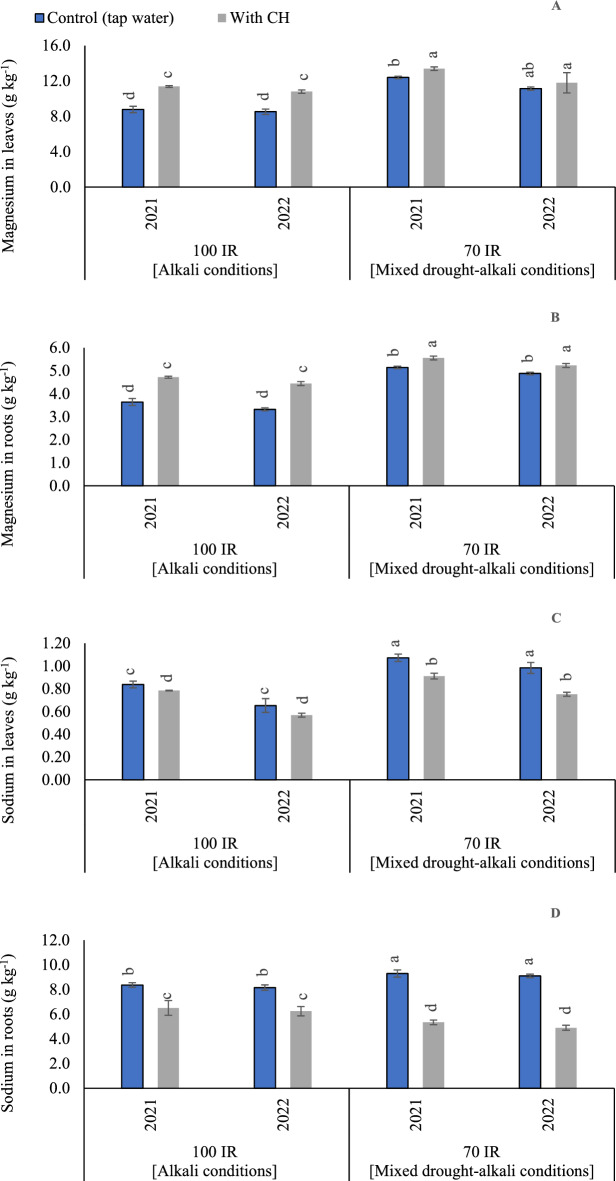
The solitary and interaction effects of CH and IR on (**A**) Magnesium content in leaves, (**B**) Magnesium content in roots, (**C**) and Sodium content in leaves, and (**D**) Sodium content in roots at two seasons of 2021/2022. Vertical bars represent ± standard error (SE) of the means. Different letters in the bars indicate significant differences between treatments at P ≤ 0.05 level by the Tukey’s test. Abbreviations: Control (tap water applications); CH (foliar chitosan applications at a rate of 500 mg L^-1^); IR100% (100% of the irrigation water requirements- represent alkali condition); IR 70% (70% of the irrigation water requirements- represent mixed drought-alkali conditions).

The results of variance analysis, showed that the sole and interaction influence of CH and IR on Mg contents in peanut leaves were significant in the first and second seasons. The *p* values for CH were recorded (< 0.001 and 0.001) in the first and second seasons, respectively. Also, *p* values for IR were recorded (< 0.001 and 0.001) in the first and second seasons, respectively. Moreover, the interaction between CH and IR was recorded (< 0.001 and 0.001) in the first and second seasons, respectively. By comparing alkali and mixed drought-alkali conditions in (Fig. 4A), adopting mixed drought-alkali conditions, results in significant increases for Mg concentrations in the peanut leaves in the two growing seasons. In this sense, when adopting control limited irrigation treatment (mixed drought-alkali conditions), Mg content was increased by 40.9 and 30.6% compared to control alkali conditions, in the first and second seasons, respectively. Likewise, relative to control CH under alkali conditions, Mg contents increased by 17.5 and 9.3% for control CH under mixed drought-alkali conditions, in the first and second seasons, respectively.

#### Mg concentrations in peanut roots

The ANOVA findings showed that in the first and second seasons, the sole and interaction influence of CH and IR on Mg contents in root were significant (p < 0.05). The obtained results in (Fig. 4B) showed that the maximum Mg contents in roots were observed by applying foliar applications of CH under mixed drought-alkali conditions in the two growing seasons. While the lowest Mg concentrations in root were recorded by applying full irrigation requirements under alkali conditions without CH applications.

#### Na concentrations in peanut leaves

Based on the results of variance analysis, the sole and interaction effect of CH and IR on Na contents in leaves were significant (p < 0.05) in the two growing seasons. The *p* values for CH were recorded (< 0.001 and 0.001) in the first and second seasons, respectively. On the side, *p* values for IR were recorded (< 0.001) in the first and second seasons. While the interaction between CH and IR was recorded (*p* < 0.001) in the first season and (*p* = 0.001) in the second season. As presented in (Fig. 4C), the adoption of mixed drought-alkali conditions was attained the highest Na concentrations in leaves. The obtained results showed that by adopting 100% IR with applying tap water applications under mixed drought-alkali conditions, Na contents increased by (21.5%) compared to control alkali treatment in the first season. While, in second season by adopting 100% IR with applying tap water applications under mixed drought-alkali conditions, Na contents decreased by (33.7%) compared to control alkali treatment. On the other hand, relative to control CH under alkali conditions, Na contents increased by 16.6 and 31.6% for control CH under mixed drought-alkali conditions, in the first and second seasons, respectively.

#### Na concentrations in peanut roots

Based on the results of variance analysis, the sole effect of CH and IR on Na contents in roots was significant (p < 0.05) in the two growing seasons. While the interaction between CH and IR had a non-significant influence in the first season and had a significant influence in the second season. The *p* values for CH were recorded (0.001 and 0.020) in the first and second seasons, respectively. On the side, *p* values for IR were recorded (< 0.001) in the first and second seasons. While the interaction between CH and IR was recorded (*p* = 0.097) in the first season and (*p* < 0.001) in the second season. The impacts of the CH applications and IR (100 and 70%), on the Na contents, are presented in (Fig. 4D). By comparing the treatments under alkali and mixed drought-alkali, the Na contents in roots were significantly decreased by applying CH applications in the two growing seasons. The obtained results showed that under alkali conditions, by applying CH applications, Na contents were decreased by (22.6 and 24.4%) compared to tap water applications in the first and second seasons, respectively. While, under mixed drought-alkali conditions, by applying CH applications, Na contents were decreased by (43.0 and 46.2%) compared to tap water applications in the first and second seasons, respectively.

#### Fe concentrations in peanut leaves

**Figure 5:**
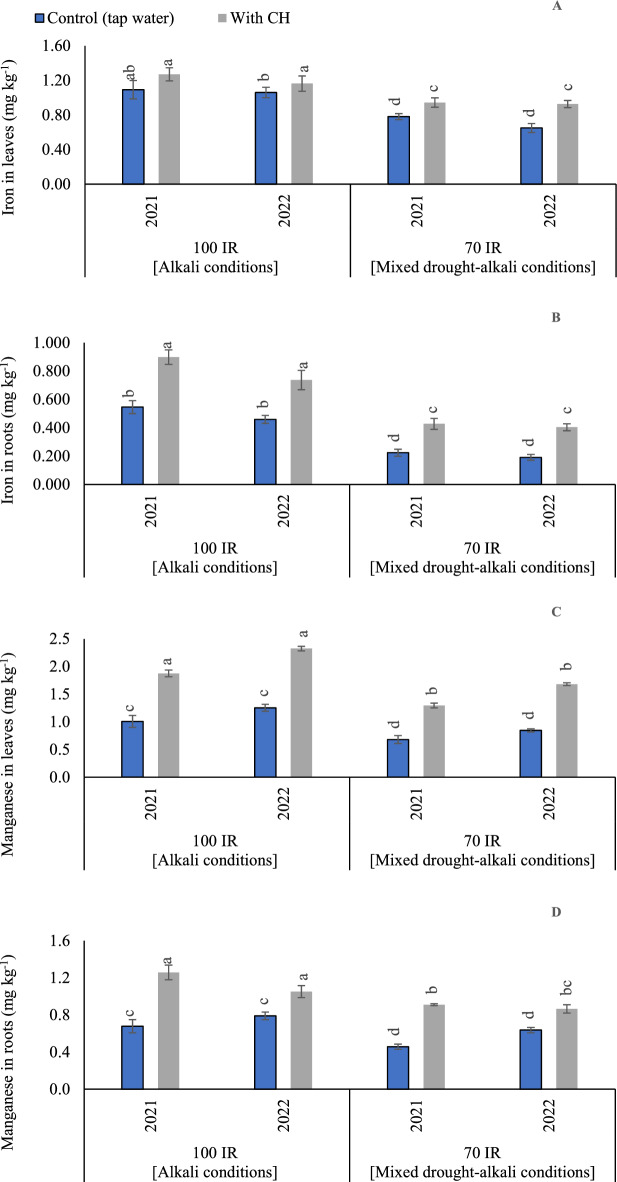
The solitary and interaction effects of CH and IR on (**A**) Iron content in leaves, (**B**) Iron content in roots, (**C**) Manganese content in leaves, and (**D**) Manganese content in roots at two seasons of 2021/2022. Vertical bars represent ± standard error (SE) of the means. Different letters in the bars indicate significant differences between treatments at P ≤ 0.05 level by the Tukey’s test. Abbreviations: Control (tap water applications); CH (foliar chitosan applications at a rate of 500 mg L^−1^); IR100% (100% of the irrigation water requirements- represent alkali condition); IR 70% (70% of the irrigation water requirements- represent mixed drought-alkali conditions).

Based on ANOVA results, the sole influence of examined CH and IR on Fe content was significant (*p* < 0.05) in the two growing seasons. The *p* values for CH were recorded (0.001) in the first and second seasons. On the side, *p* values for IR were recorded (0.003 and < 0.001) in the first and second seasons, respectively. While the interaction between CH and IR was recorded (*p* = 0.074) in the first season and (*p* = 0.001) in the second season. By comparing the treatments under alkali and mixed drought-alkali, the Fe contents were increased under alkali conditions in the two growing seasons (Fig. 5A). Relative to control mixed drought-alkali conditions, Fe contents increased by 39.7 and 63.1% for control alkali conditions, in the first and second seasons, respectively. Likewise, relative to control CH under mixed drought-alkali conditions, Fe contents increased by 35.1 and 27.7% for control CH under alkali conditions, in the first and second seasons, respectively.

#### Fe concentrations in peanut roots

Based on the results of variance analysis, the sole and interaction effect of CH and IR on Fe contents in roots were significant (p < 0.05) in the two growing seasons. The *p* values for CH were recorded (< 0.001) in the first and second seasons. Also, *p* values for IR were recorded (< 0.001) in the first and second seasons. While the interaction between CH and IR was recorded (*p* = 0.002) in the first season and (*p* = 0.041) in the second season. The effects of the CH applications and IR (100 and 70%), on the Fe contents, are presented in (Fig. 5B). By comparing the treatments under alkali and mixed drought-alkali, the Fe contents were significantly increased under the alkali conditions in the two growing seasons than the mixed drought-alkali conditions. Additionally, Fe contents were significantly enhanced by applying CH applications. The obtained results showed that by adopting 100% IR without applying CH applications under alkali conditions, Fe contents increased by (143.8 and 140.3%) compared to control mixed drought-alkali treatment. While, by applying CH applications under alkali conditions, Fe contents increased by (107.7 and 82.4%) compared to mixed drought-alkali conditions in the first and second seasons, respectively.

#### Mn concentrations in peanut leaves

The ANOVA findings showed that in the first and second seasons, the sole and interaction influence of CH and IR on Mn contents in leaves were significant (p < 0.05). The *p* values for CH, IR, and their interaction were recorded (< 0.001 and 0.001) in the first and second seasons, respectively. By comparing alkali and mixed drought-alkali conditions in (Fig. 5C), adopting mixed drought-alkali conditions, results in significant decreases for Mn concentrations in the peanut leaves in the two growing seasons. In this sense, when adopting control mixed drought-alkali conditions, Mn content was decreased by 30.0 and 38.5% compared to control alkali conditions, in the first and second seasons, respectively. Likewise, relative to control CH under mixed drought-alkali conditions, Mn contents decreased by 31.6 and 26.1%, in the first and second seasons, respectively.

#### Mn concentrations in peanut roots

The ANOVA findings indicated that the sole influence of examined CH and IR on Mn content in roots was significant (*p* < 0.05) in the two growing seasons. The *p* values for CH were recorded (0.003) in the first and second seasons. Also, *p* values for IR were recorded (< 0.001) in the first and second seasons. While the interaction between CH and IR had non-significant influence (*p* = 0.051 and 0.056), in the first and second seasons, respectively. The Mn content in the root was raised by 24.0 and 23.9% for CH treatments under alkali conditions compared to control treatment (tap water), in the first season and second seasons, respectively (Fig. 5D). Likewise, Mn content in root was raised by 46.2 and 27.3% for CH treatments under mixed drought-alkali conditions compared to control treatment (tap water), in the first season and second seasons, respectively. The highest Mn content in the root was significantly recorded by applying the foliar applications of CH under alkali conditions. Furthermore, the lowest Mn content in root was significantly obtained by applying tap water under mixed drought-alkali conditions in the first season and second seasons, respectively.

#### Zn concentrations in peanut leaves

**Figure 6:**
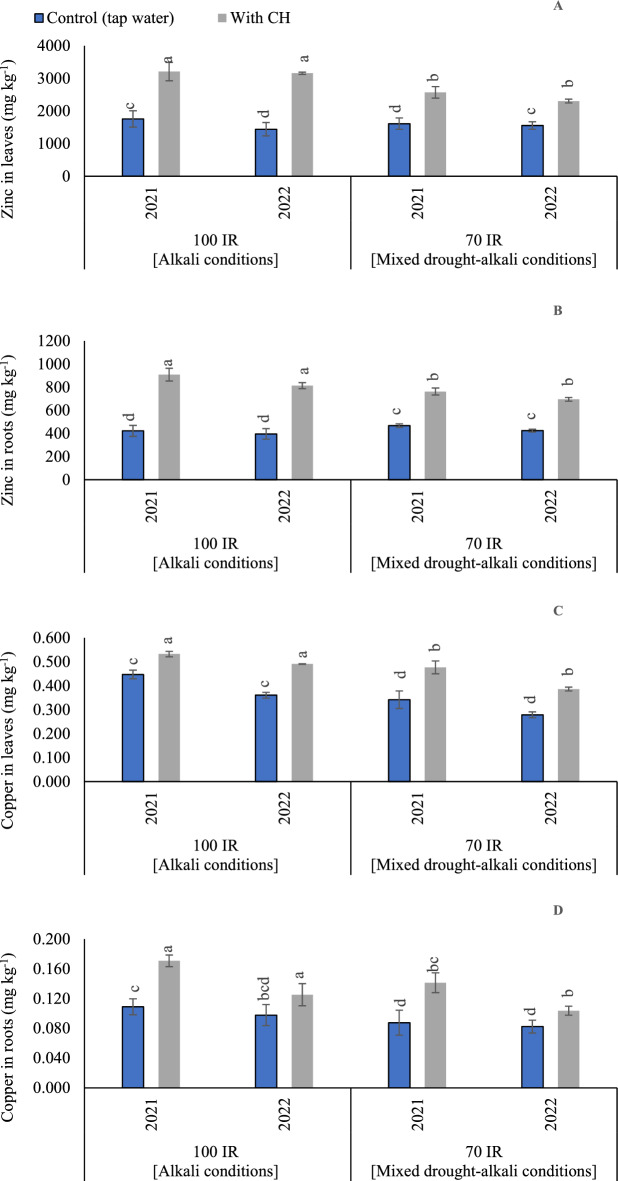
The solitary and interaction effects of CH and IR on (**A**) Zinc content in leaves, (**B**) Zinc content in roots, (**C**) Copper content in leaves, and (**D**) Copper content in roots at two seasons of 2021/2022. Vertical bars represent ± standard error (SE) of the means. Different letters in the bars indicate significant differences between treatments at P ≤ 0.05 level by the Tukey’s test. Abbreviations: Control (tap water applications); CH (foliar chitosan applications at a rate of 500 mg L^−1^); IR100% (100% of the irrigation water requirements- represent alkali condition); IR 70% (70% of the irrigation water requirements- represent mixed drought-alkali conditions).

Based on the results of variance analysis, the sole and interaction effect of CH and IR on Zn contents in leaves were significant (p < 0.05) in the two growing seasons. The *p* values for CH were recorded (0.004 and 0.002) in the first and second seasons, respectively. On the side, *p* values for IR were recorded (< 0.001) in the first and second seasons. While the interaction between CH and IR was recorded (*p* = 0.004) in the first season and (*p* < 0.001) in the second season. As presented in (Fig. 6A), the adoption of mixed drought-alkali conditions was pronounced with the foliar CH in attaining the highest Zn concentrations in leaves. Therefore, these findings emphasize the importance of applying CH applications, especially under limited irrigation conditions. This approach results the highest accumulations of Zn concentrations, resulting in enhanced peanut tolerance against alkaline stress impacts. The gained results showed that by adopting 100% IR without applying CH applications under alkali conditions, Zn contents increased by (8.1%) compared to control mixed drought-alkali treatment in the first season. While, in second season by adopting 100% IR without applying CH applications under alkali conditions, Zn contents decreased by (7.5%) compared to control mixed drought-alkali treatment. On the other hand, by applying CH applications under alkali condition, Zn contents increased by (24.8 and 37.0%) compared to mixed drought-alkali conditions in the first and second seasons, respectively. On the other side, the lowest Zn concentrations were recorded under alkali and mixed drought-alkali conditions.

#### Zn concentrations in peanut roots

Based on the results of variance analysis, the sole and interaction effect of CH and IR on Zn contents in roots were significant (p < 0.05) in the two growing seasons. The *p* values for CH were recorded (0.019 and 0.003) in the first and second seasons, respectively. On the side, *p* values for IR were recorded (< 0.001) in the first and second seasons. While the interaction between CH and IR was recorded (*p* = 0.001) in the first season and (*p* = 0.0002) in the second season. The impacts of the CH applications and IR (100 and 70%), on the Zn contents, are presented in (Fig. 6B). By comparing the treatments under alkali and mixed drought-alkali, the Zn contents were significantly increased by applying CH applications in the two growing seasons. The obtained results showed that by adopting 100% IR and applying tap water applications under alkali conditions, Zn contents decreased by (10.5 and 7.4%) compared to control mixed drought-alkali treatment. While, by applying CH applications under alkali condition, Zn contents increased by (19.1 and 16.9%) compared to mixed drought-alkali conditions in the first and second seasons, respectively.

#### Cu concentrations in peanut leaves

Based on the results of variance analysis, the sole and interaction effect of CH and IR on Cu concentrations in leaves were significant (p < 0.05) in the two growing seasons. The *p* values for CH were recorded (0.001 and 0.044) in the first and second seasons, respectively. On the side, *p* values for IR were recorded (0.003 and 0.001) in the first and second seasons, respectively. While the interaction between CH and IR was recorded (*p* = 0.045) in the first season and (*p* < 0.001) in the second season. By comparing the treatments under alkali and mixed drought-alkali, the Cu contents were significantly increased under the alkali conditions or applied CH applications in the two growing seasons than the mixed drought-alkali conditions (Fig. 6C). The obtained results showed that under alkali conditions by adopting 100% IR and applying CH applications, Cu contents increased by (16.1 and 26.5%) compared to control alkali treatment (tap water). Likewise, by applying CH applications under mixed drought- alkali condition, Cu contents increased by (28.3 and 27.8%) compared to control mixed drought-alkali conditions in the first and second seasons, respectively.

#### Cu concentrations in peanut roots

Based on ANOVA results, the sole influence of examined CH and IR on Fe content was significant (*p* < 0.05) in the two growing seasons. While the interaction between CH and IR had a non-significant influence (*p* > 0.05) in the two growing seasons. The *p* values for CH were recorded (< 0.001 and 0.013) in the first and second seasons, respectively. On the side, *p* values for IR were recorded (< 0.001) in the first and second seasons. While the interaction between CH and IR was recorded (*p* = 0.380) in the first season and (*p* = 0.109) in the second season. The obtained findings in (Fig. 6D) showed that under alkali conditions by adopting 100% IR and applying CH applications, Cu contents in roots were increased by (36.3 and 21.6%) compared to control alkali treatment (tap water). Likewise, by applying CH applications under mixed drought- alkali condition, Cu contents were increased by (37.6 and 21.2%) compared to control mixed drought-alkali conditions in the first and second seasons, respectively.

### The solitary and interaction effects of CH and IR under alkali and mixed drought-alkali conditions on the agronomic traits and soil pH

Details of solitary and interaction effects for CH and IR under alkali and mixed drought-alkali conditions on (Shoot fresh weight, weight of pods, and seed index at two growing seasons), are provided in Supplementary Table S1. While the detailed list of measured traits (root length and soil pH) is given in Supplementary Table S2.

### The solitary and interaction effects of CH and IR under alkali & mixed drought-alkali conditions on

#### Seeds yield

**Figure 7:**
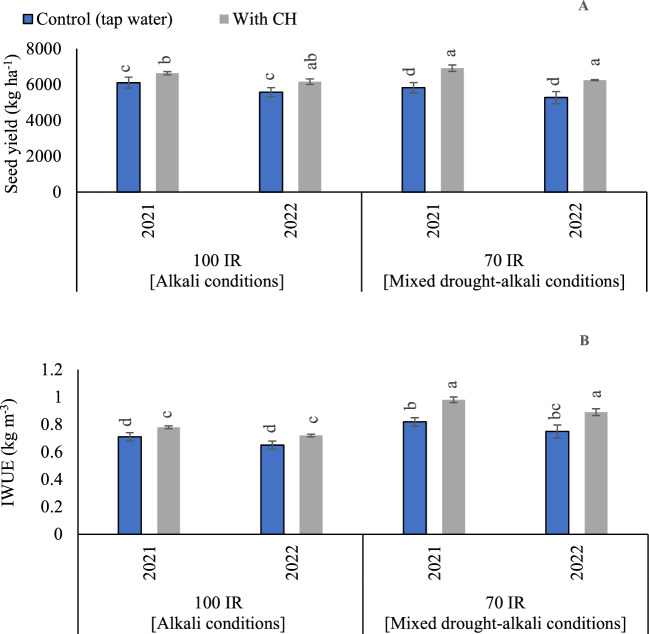
The solitary and interaction effects of CH and IR on (**A**) seeds yield and (**B**) Irrigation water use efficiency (IWUE) of the peanut crop at two seasons of 2021/2022. Vertical bars represent ± standard error (SE) of the means. Different letters in the bars indicate significant differences between treatments at P ≤ 0.05 level by the Tukey’s test. Abbreviations: Control (tap water applications); CH (foliar chitosan applications at a rate of 500 mg L^−1^); IR100% (100% of the irrigation water requirements- represent alkali condition); IR 70% (70% of the irrigation water requirements- represent mixed drought-alkali conditions).

Based on the results of variance analysis (Tables 4 and 5), the sole impact of IR had non-significant influence (*p* > 0.05) in the two growing seasons. While the sole CH and the interaction impacts of examined (CH and IR) were significant (p < 0.05) in the two growing seasons. The *p* values for CH were recorded (0.946 and 0.114) in the first and second seasons, respectively. On the side, *p* values for IR were recorded (< 0.001) in the first and second seasons. While the interaction between CH and IR was recorded (*p* = 0.005) in the first season and (*p* = 0.017) in the second season. The findings in (Fig. 7A), demonstrated that the CH applications worked as a cofactor for enhancing peanut seed yield under alkali and mixed drought-alkali conditions. Relative to tap water applications under 100% IR (alkali condition), the obtained results showed that by applying CH applications, peanut yield was increased by (8.6 and 10.5%), in the first and second seasons, respectively. Likewise, relative to tap water applications under 70% IR (mixed drought-alkali conditions), the obtained results showed that by applying CH applications, peanut yield was increased by (18.7 and 18.5%), in the first and second seasons, respectively. The highest peanut yield was recorded by applying foliar applications of CH under 70% IR (mixed drought-alkali conditions) in the first and second seasons, respectively. However, that was significantly equaled by applying CH under 100% IR (alkali condition) in the second season. On the other hand, the obtained results showed that the lowest peanut yield was significantly obtained by applying tap water applications under mixed drought-alkali conditions.

#### IWUE

Based on the results of variance analysis, the sole and interaction effect of CH and IR were significant (p < 0.05) in the two growing seasons. The *p* values for CH were recorded (< 0.001) in the first and second seasons. Also, *p* values for IR were recorded (< 0.001) in the first and second seasons. While the interaction between CH and IR was recorded (*p* = 0.002) in the first season and (*p* = 0.005) in the second season. As can be seen in (Fig. 7B), by comparing the treatments under alkali and mixed drought-alkali, IWUE was increased and attained the highest values under the mixed drought-alkali conditions condition in the two growing seasons. IWUE values for peanut were increased from (0.71 and 0.65 kg m^−3^) for the (tap water × alkali condition) to (0.98 and 0.89 kg m^−3^) for (CH × mixed drought-alkali conditions condition) in the first and second seasons, respectively.

## Discussion

Drought and alkali stresses affect peanut plantations in arid and semi-arid regions, where approximately 70% of peanut cultivation occurs^63^. The majority of research focuses on the effects of drought or alkali stress on peanut plants, rather than both simultaneously. As a result, the present study endeavored to address the limited understanding of plant responses, particularly in the context of combined drought-alkali stresses. In situations where effective administration and proper recognition are critical, there are viable strategies that can be implemented to increase and sustain productivity.

### Monitoring the physio-biochemical traits of peanut under alkali and mixed drought-alkali stresses

The results of this investigation demonstrated that although mixed drought-alkali stresses resulted in reduced values for total chlorophyll and carbohydrates in peanut plants, the concentration of RWC and proline exhibited an improvement in comparison to the effect of alkaline stress alone during both growing seasons. The observed decline in RWC was anticipated in alkaline environments, as prior research has documented that alkaline soils have unfavorable characteristics including a high infiltration rate, poor structure, and low holding capacity^64^. While this rise in RWC under mixed drought-alkali environments appears to be attributable to A) an elongation of the roots, which facilitates the absorption of adequate quantities of water. This assimilation resulted in an enhancement in the quantity of water that could be absorbed by roots. Dien et al.^65^ documented in this regard that plants with greater root systems are capable of assimilating a greater quantity of water from the soil when subjected to stressful conditions, thereby enhancing their tolerance to stress. An additional factor that contributed to the observed enhancement in RWC under mixed drought-alkali conditions was B) the accumulation of proline. Previous research has documented that plants under stress tend to increase their production and accumulation of osmolytes, such as proline^66–69^. Proline accumulation in response to abiotic stresses not only facilitates osmotic adjustment and redox balance, but also contributes to the stabilization of cell membranes and the preservation of the turgidity of plant leaves, the authors continued. Previous research has suggested that an increase in stress intensity may cause plants to produce proline at an accelerated rate, accompanied by a decrease in chlorophyll content^5,15^. Proline accumulation under mixed drought-alkali conditions is regarded as a crucial tolerance mechanism employed by plants to withstand stressors. Furthermore, the cultivar's notable capacity for proline accumulation may indicate a robust resistance to combined drought and alkali stresses, as supported by Ref.^65^. While it was previously established that the Giza 6 variety of peanut is drought-tolerant variety^22^, information regarding its tolerance to alkaline or mixed drought-alkali stresses was unavailable. Proline accumulations allow intracellular water potential to be maintained and water's solvent properties to be altered in response to stress^5,70^, thereby enabling osmotic adjustment. In addition, proline accumulates in response to stress, acts as a protective agent for enzymes, and stabilizes the structure of both proteins and cell membranes^65,71,72^. On the other hand, the results indicated that carbohydrates exhibited the lowest value under mixed drought-alkali stresses in both seasons. In relation to the decrease in carbohydrates during mixed drought-alkali stresses, a study by Ghouili et al.^71^, demonstrated that the accumulation of various carbohydrates is contingent upon the level of stress, plant species, plant organs, and cultivar employed. Moreover, proline levels were found to be elevated when exposed to alkali, and previous research has documented an antagonistic relationship between proline and carbohydrates^73,74^. Furthermore, Guo et al.^75^ noted that it appears plants under stress conditions obtain the energy necessary to maintain cellular osmotic homeostasis through the breakdown of polysaccharides.

### Monitoring the nutritional status of peanut under alkali and mixed drought-alkali stresses

The findings indicate that in the presence of combined drought and alkali stresses, plants exhibit a tendency to develop tolerance by increasing the concentrations of certain nutrients in their leaves and roots. The findings indicate that peanuts subjected to mixed drought-alkali stresses exhibited higher concentrations of (N, K, Ca, Mg, and Na) in comparison to alkali conditions. This was not the case with regards to (P, Fe, Mn, and Cu nutrients). This study ascribed the observed increases in (N, K, Ca, and Mg) to the presence of Na, suggesting that this strategy functions as a defensive mechanism. Reducing the quantity of water applied resulted in higher concentrations of nutrients, including Ca, Na, and Mg, which was reflected in an increase in the quantities absorbed by these elements. Cell wall structure is impaired when subjected to a combination of drought and alkali stresses and elevated Na concentrations^76^. During this period, Ca was advantageous for improved hydration status, enhanced structural and functional integrity of cell membranes, and regulated ion-exchange behavior^77^. Moreover, Yokoi et al.^78^ noted that Ca induces stress responses through the direct inhibition of Na influx, a property that would have proven beneficial in the given circumstances. N and Mg were found to be involved in chlorophyll synthesis in the presence of stress^79,80^. Hasanuzzaman et al.^81^ state that increased K concentrations assist plants in regulating osmotic balance and maintaining ion homeostasis. On the other side, the obtained results demonstrated a decrease in (P, Fe, Mn, and Cu) absorption in mixed drought-alkali environments. According to the findings of Keisham et al.^82^, P fixed under stressful conditions due to the formation of insoluble precipitates with Ca, such as hydroxyapatite^83^, which inhibit the assimilation of P ions by the roots of plants. Guo et al.^75^ additionally documented a reduction in P absorption when exposed to alkali conditions. While the degradation of the availability and subsequent absorption of (Fe, Mn, and Cu) was ascribed to the influence of higher pH level^84^.

It is noteworthy to mention that the findings suggest that when plants are subjected to a combination of drought and alkali stresses, they exhibit a tendency to alter the accumulation of nutrients between their roots and leaves. It was established that leaf accumulation of (K, Ca, Fe, Zn, and Cu) is more prevalent in plants compared to root accumulation. Na was evidently preferable for accumulation in roots. This study ascribed this characteristic to the peanuts' (cultivar Giza 6) remarkable resilience under adverse growth conditions. As illustrated in (Figs. 3A,C and 6A), increasing the accumulations of (K, Ca, and Zn) in the leaves rather than roots assisted in maintaining a secure water status. Moreover, elevated concentrations of (Fe, Zn, and Cu) in the foliage sustained the enzymatic and chlorophyll degradation processes, whereas Na was absent in the roots. Regarding this matter, Khan et al.^85^ demonstrated that certain plants have the capacity to release Na^+^ ions from within their cells (extracellular area). This is regarded as a significant physiological mechanism employed by plants to alleviate the detrimental effects of stress.

### The changing in nutrient homeostasis as a consequence of CH under alkali and mixed drought-alkali conditions

Multiple studies have demonstrated that CH is capable of eliciting a wide variety of defense responses in response to abiotic stressors^86–88^. Nevertheless, there is a scarcity of research examining the reflecting effects that occur on nutrient accumulations as a result of CH applications in alkali and mixed drought-alkali environments. As a result, this study attempts to provide a comprehensive overview of the nutrient homeostasis changes induced by CH under these stresses in this paragraph.

According to the results obtained, CH altered the nutritional composition of peanut leaves and roots by causing accumulations of macronutrients and micronutrients. When CH applications were applied under alkali conditions, the accumulations of (P, Ca, Fe, Mn, Zn, and Cu) in leaves and roots were maximized, while the accumulation of Na was reduced to a lower value. It appears that CH enhanced root length under these growth conditions, thereby increasing the plant's capacity to absorb nutrients and water^89^. Furthermore, it has been observed that in soil solutions with high alkalinity, Ca concentrations predominate^83,90^. Consequently, the application of CH applications facilitated Ca influx into the cytosol, as documented by Mukarram et al.^91^, and contributed to the reinforcement of cell structure^92^. Furthermore, the application of CH enhanced osmotic adjustment through the accumulation of more Zn, which led to an increase in RWC. Maintain the carbon balance by increasing P accumulation, which is essential for the synthesis of carbohydrates in response to stress^32^. Moreover, CH applications maintain water status and photosynthesis components (chlorophyll contents) through the accumulation of Fe and Mn, where Fe and Cu are essential for the photosynthesis process and nitrogen reduction^93,94^. Hebbern et al.^95^ demonstrated the significance of Mn in the process of cuticular wax deposition on leaves, resulting in increased water content within tissues (RWC). Furthermore, CH applications appear to have played a role in the reduction of Na accumulations by reducing Na accumulations in leaves and roots, thereby protecting against an increase in Na hazard.

On the other side, applied CH under conditions of mixed drought-alkali stress led to the greater accumulations of (N, K, and Mg) in peanut leaves and roots compared to alkali conditions, by increasing the stress intensity. While Na accumulations recorded the minimum significant value in peanut leaves and roots. To elucidate these findings, we discovered that the significance of preserving the aforementioned nutrients' accumulations is well-known and has been extensively discussed in the preceding paragraphs. Our focus was drawn to the following: (A) the preservation of preventing or minimizing Na accumulations, particularly in the leaves, through the reduction of Na flux from the soil or roots, which is crucial for stress resistance. (B) the emphasis that peanuts placed on (N, K, and Mg) accumulations in leaves and roots in response to mixed drought-alkali stresses (greater stress intensity) as opposed to (P, Ca, Fe, Mn, Zn, and Cu). The aforementioned findings pertained to the capacity of peanuts to modify their nutrient accumulations and prioritization in response to stress conditions, thereby ensuring their resistance, which are consistent with findings from Ref.^95^. Furthermore, the findings of this study indicate that in order to alleviate the severity of stress, it is crucial to employ (P, Ca, Fe, Mn, Zn, and Cu) nutrients during alkali growth conditions, and (N, K, and Mg) nutrients in combination with CH during mixed drought-alkali conditions. Comparably, when Farouk & Amany^96^ examined the effects of applying CH under stressful conditions on cowpea, they observed increases in (N and K) in the shoots. Hidangmayum et al.^32^, on the other hand, suggested that increases in (N and K) in the leaves contribute to an increase in the number of chloroplasts per cell and enhance chlorophyll synthesis, thereby mitigating the negative effects. Abou El-Enin et al.^97^ found that foliar nano CH-loaded nitrogen increases the productivity of intercropped maize and soybean in calcareous soil. Under drought stress, both Bukhari et al.^98^ and Ali et al.^99^ discovered beneficial effects of (N and K) applications and various concentrations combined with CH. To confirm or refute the aforementioned hypotheses, however, additional research is necessary under alkali and mixed drought-alkali stress conditions in the future.

### Feasibility of CH application to boost peanut yield and IWUE under alkali and mixed drought-alkali conditions

Based on the results obtained, CH applications under mixed drought-alkali conditions produced the highest seed yield and IWUE of the peanuts while requiring the least amount of water to be applied. The advantages of utilizing a mild drought irrigation level in a combination with CH under mixed stress growth conditions were attributed to this concern. In the given conditions, peanut plants were subjected to a specific level of dehydration stress, which triggered a cascade of subsequent effects. Stresses were alleviated by the subsequent increases in proline and nutrient uptake such as (N, K, and Mg). This study ascribed this concern to the tolerance capacity exhibited by plants. Previous studies^25,100–102^ have documented that peanut plants can withstand higher pH levels by increasing their root secretions, which allows them to withstand adverse growing conditions. These organic secretions can function as a buffer, enabling plants to withstand environmental fluctuations and preserve intracellular pH stability and ion balance. Therefore, it appears that the marginal reduction in soil pH, as illustrated in (Supplementary Table S2), resulted in an enhance in the nutrient absorption. These enhancements further improved (shoot fresh weight, pod weight, root length, and seed index), and ultimately led to the highest seed yield and IWUE. These results are consistent with those reported by Refs.^17,32,37,86^.

## Conclusion

The present study observed that peanuts exhibited diverse responses to stress induced by alkali and mixed drought-alkali conditions. In contrast to the alkali stress condition, (carbohydrates and total chlorophyll) decreased significantly under mixed drought-alkali stress conditions. Under conditions of mixed drought-alkali stress, plants accumulated a greater quantity of (K, Ca, Fe, Zn, and Cu) in their leaves as opposed to their roots, according to this study. On the other hand, Na was evidently preferred for accumulation in roots. This could potentially be the peanut plant's way of defending its leaves against the detrimental effects of Na stress. As an osmotic protectant, peanut plants were able to amass elevated levels of proline in response to chitosan applications, which averted severe damage caused by combined drought and alkali stresses. Chitosan accumulation increases the accumulation of (N, K, and Mg) in leaves and roots under mixed drought-alkali stress conditions, as opposed to (P, Ca, Fe, Mn, Zn, and Cu) under alkali stress conditions. The findings of this study indicate that peanut plants implemented distinct strategies in reaction to stresses characterized by varying levels of nutrient accumulation. This research suggests that supplementary applications of chitosan (500 mg L^−1^) applied foliarly to peanut plants cultivated in alkali soil at a rate of 70% of irrigation water requirements can increase proline, (N, K and Mg), pod weight, seed yield, and irrigation water use efficiency.

### Supplementary Information

Supplementary Tables.

## Data Availability

The presented datasets during the current study available from the corresponding authors on reasonable request.
