# Supplementary material for: Monitoring plant responses in field-grown peanuts exposed to exogenously applied chitosan under full and limited irrigation levels
Source: Sci Rep. 2024 Mar 15;14:6244. doi: 10.1038/s41598-024-56573-6 (PMC10940646; doi:10.1038/s41598-024-56573-6)
Supplement: Supplementary file 1 — Supplementary Tables. [file 41598_2024_56573_MOESM1_ESM.pdf]

Table S1 The solitary and interaction effects of CH and IR on shoot fresh weight, weight of pods plant^{-1}, and seed index (weight of 10 seeds) at two seasons of 2021/2022.

Investigated parameters			Growing season 2021	Growing season 2022
Shoot fresh weight (kg ha^{-1})	100 % IR [Al- con]	Control (tap water)	27460±2591c	26356±2450c
		With CH	30400±1306b	29120±1306b
	70% IR [Mix-con]	Control (tap water)	26400±2788cd	25320±2486cd
		With CH	35160±2175a	33720±2917a
	p-value	Irrigation levels (IR)	0.331	0.335
		Chitosan (CH)	<0.001	0.001
		IR × CH	0.012	0.011
Weight of pods (kg ha^{-1})	100 % IR [Al- con]	Control (tap water)	6724±330cd	6297±246cd
		With CH	7312±93b	6760±270b
	70% IR [Mix-con]	Control (tap water)	6416±318d	5939±318d
		With CH	7630±264a	7153±132a
	p-value	Irrigation levels (IR)	0.925	0.816
		Chitosan (CH)	<0.001	<0.001
		IR × CH	0.007	0.001
Seed index (g)	100 % IR [Al- con]	Control (tap water)	8.6±0.84cd	7.83±0.75cd
		With CH	10.66±0.96b	9.7±0.82b
	70% IR [Mix-con]	Control (tap water)	7.71±0.82d	7.0±0.82d
		With CH	11.36±1.2a	10.3±1.1a
	p-value	Irrigation levels (IR)	0.258	0.343
		Chitosan (CH)	<0.001	0.001
		IR × CH	0.036	0.040

Represented data are the mean value± standard error (SE). Different lowercase letters above error bars indicate statistically significant differences ($p < 0.05$). Abbreviations: with CH (foliar chitosan applications at a rate of 500 mg L^{-1}); IR100% (100% of the irrigation water requirements- represent alkali condition); IR 70% (70% of the irrigation water requirements- represent mixed drought-alkali conditions); Al- con (represent alkali conditions); Mix-con (represent mixed drought-alkali conditions).

Table S2 The solitary and interaction effects of CH and IR on root length and soil pH at two seasons of 2021/2022.

Investigated parameters			Growing season 2021	Growing season 2022
**Root length (cm)**	**100 % IR [Al- con]**	**Control (tap water)**	9.3±0.057d	9.6±0.057d
		**With CH**	13.4±0.5b	13.7±0.5b
	**70% IR [Mix-con]**	**Control (tap water)**	11.3±0.57c	11.7±0.57c
		**With CH**	16.4±0.25a	16.9±0.25a
	***p*-value**	**Irrigation levels (IR)**	<0.001	<0.001
		**Chitosan (CH)**	<0.001	<0.001
		**IR × CH**	0.003	0.003
**Soil pH**	**100 % IR [Al- con]**	**Control (tap water)**	7.78±0.01a	7.93±0.016a
		**With CH**	7.73±0.01b	7.88±0.01b
	**70% IR [Mix-con]**	**Control (tap water)**	7.54±0.01c	7.71±0.012c
		**With CH**	7.47±0.01d	7.64±0.01d
	***p*-value**	**Irrigation levels (IR)**	<0.001	<0.001
		**Chitosan (CH)**	<0.001	<0.001
		**IR × CH**	0.01	0.01

Represented data are the mean value± standard error (SE). Different lowercase letters above error bars indicate statistically significant differences ($p < 0.05$). Abbreviations: with CH (foliar chitosan applications at a rate of 500 mg L^{-1}); IR100% (100% of the irrigation water requirements- represent alkali condition); IR 70% (70% of the irrigation water requirements- represent mixed drought-alkali conditions); Al- con (represent alkali conditions); Mix-con (represent mixed drought-alkali conditions).

.
